# A Barrier to Understanding Teratogenicity: The Critical Periods of Sensitivity for Most Structural Birth Defects Precede the Established Hemochorial Placenta

**DOI:** 10.1002/bdr2.2532

**Published:** 2025-09-29

**Authors:** Matthew A. Nangle, Khush Shah, Sathish Kumar, Robert J. Lipinski

**Affiliations:** ^1^ Department of Comparative Biosciences, School of Veterinary Medicine University of Wisconsin‐Madison Madison Wisconsin USA; ^2^ Department of Medicine Lake Erie College of Osteopathic Medicine at Seton Hill Greensburg Pennsylvania USA

**Keywords:** birth defects, critical periods, placenta, teratogens

## Abstract

**Background:**

Teratogens and other environmental factors influence human birth defect risk, but our understanding of how they reach the developing conceptus is surprisingly limited. The placenta is often invoked as a key mediator of teratogenicity by acting as a physical barrier that can block or regulate the transfer of harmful substances to the embryo or fetus.

**Methods:**

In this review, we compare the timing of teratogen susceptibility with the development of the placenta. Teratogenicity data from multiple published studies were plotted on a unified multi‐species developmental timeline to relate findings from animal models to human developmental timing.

**Results:**

The critical periods for most teratogen‐induced structural birth defects, including fetal alcohol syndrome‐related defects, neural tube defects, orofacial clefts, and limb malformations translate to the 3rd to 6th week of human embryonic development, while the human hemochorial placenta matures later, between 8 and 12 weeks of pregnancy.

**Conclusions:**

This developmental chronology challenges the seemingly pervasive notion that placental transfer capacity plays a major role in mediating teratogenicity and highlights the need to further investigate the barrier capacity of the structures that surround and protect the developing embryo (e.g., trophoblast, yolk sac) prior to formation of the definitive placenta, and when the embryo is most sensitive to teratogenic insult.

## 1 Introduction

Birth defects occur in approximately 1 in 33 births and are a major cause of morbidity and mortality. While the majority of birth defects still cannot be attributed to a specific cause, most are thought to result from complex interactions among genetic and environmental influences (Feldkamp et al. 2017; Lipinski and Krauss 2023). The current “gene–environment” view was shaped by the arc of birth defects research, which dramatically shifted from a focus on genetics to the environment in the 1950s (Fraser 2008). Until then, the uterus was generally believed to be impervious to external influences. This view was upended by findings in experimental animal models as well as observations in children, including those with limb malformations caused by thalidomide, a drug marketed to pregnant women and later discovered to be a teratogen (Kim and Scialli 2011). Subsequently, alcohol (ethanol), retinoic acid, and other major teratogens were identified by the astute clinician approach and validated by animal model investigations (Jones and Carey 2011). In addition to these major teratogens, many dozens of environmental influences, including drugs, chemicals, and pollutants, have been identified as birth defect risk factors.

The initial view that the uterus was impervious to external influences, as well as the surprise that it was not, appears grounded in a longstanding assumption that the placenta serves as a barrier that protects the conceptus from exposure to maternally circulating xenobiotics (Koren and Ornoy 2018). The persistence and pervasiveness of this assumption are illustrated in Table 1, which presents examples of published papers in which the placenta is implicated as mediating the transfer of the most well‐studied teratogens, including thalidomide, ethanol, retinoic acid, and valproic acid. Accordingly, whether and how specific teratogens interact with the placenta has been the focus of numerous in vivo and in vitro investigations (Klein et al. 1981; Kraft et al. 1986; Asai et al. 1993; Barzago et al. 1996; Semczuk‐Sikora et al. 2010; Yuan et al. 2017).

**TABLE 1:** Selected passages invoking placenta‐mediated teratogenicity.

Publication	Year	Teratogen(s)	Salient passage
Low teratogenicity of 13‐*cis*‐retinoic acid (isotretinoin) in the mouse corresponds to low embryo concentrations during organogenesis: comparison to the all‐trans isomer (Kraft et al. 1986)	1986	Retinoic acid	Our results indicate that the low teratogenicity of 13‐*cis*‐retinoic acid in the mouse is the result of minimal placental transfer of this compound and of its 4‐oxo metabolite, which contrast sharply with the extensive placental transfer and high teratogenicity of the corresponding isomers with the all‐*trans* configuration.
Human placental transport and metabolism of all‐trans retinoic acid in vitro (Asai et al. 1993)	1993	Retinoic acid	All‐trans retinoic acid (TRA), a natural form of vitamin A, is known to be both a morphogen and a teratogen. The purpose of this investigation is to determine how the human placenta regulates and modulates the transplacental transfer and metabolism of TRA using a dual recirculating perfusion system.
Critical Periods for Prenatal Alcohol Exposure (Coles 1994)	1994	Alcohol	There is another, earlier stage—pre‐implantation—before the fertilized egg attaches, which used to be considered a safe period because the developing embryo is not yet connected to the maternal bloodstream and, therefore, does not appear to be receiving the teratogenic substance. In the 1980's, however, research revealed that these ideas may not be completely accurate. Many chemicals and organisms are able to cross the placental barrier. These teratogens also have been shown in some studies to affect the fetus during the earliest part of pregnancy that has been studied.
Placental transfer of valproic acid after liposome encapsulation during in vitro human placenta perfusion (Barzago et al. 1996)	1996	Valproic acid	Valproic acid (VPA) is an antiepileptic drug that crosses the placenta freely. Because its use in pregnancy is associated with an increased incidence of fetal malformation and toxic effects, this study was designed to check whether the placental transfer of VPA entrapped in liposomes was reduced.
Dose‐ and route‐dependent teratogenicity, toxicity, and pharmacokinetic profiles of the hedgehog signaling antagonist cyclopamine in the mouse (Lipinski et al. 2008)	2008	Cyclopamine	To our knowledge, transplacental transfer of cyclopamine has not been reported. To determine the amniotic concentration of cyclopamine relative to dam serum, we implanted OsPs dispensing 160 mg/kg/day for 31 h into pregnant dams at E8.25. Analysis of dam serum 24 h after pump implantation demonstrated a mean cyclopamine concentration of 2.26 μM, whereas the mean concentration in amniotic fluid of exposed embryos was 1.45 μM.
Of mice and children: Reminiscences of a teratogeneticist (Fraser 2008)	2008	Cortisone	More important, perhaps, is the demonstration of how an embryo's *normal* developmental pattern may influence its liability to teratogenic insult. Thus, differences in metabolism or placental transport of cortisone are not necessary to explain strain differences in liability (though they undoubtedly exist)—the difference may lie simply in the inborn developmental pattern.
Thalidomide induces limb defects by preventing angiogenic outgrowth during early limb formation (Therapontos et al. 2009)	2009	Thalidomide	A surprising feature of thalidomide is that it is not teratogenic in some animal species, particularly the mouse. However, our results clearly show that mouse blood vessels are sensitive to CPS49. One possibility is that thalidomide does not pass through the mouse placenta.
Valproic acid transfer across human placental cotyledon during dual perfusion in vitro (Semczuk‐Sikora et al. 2010)	2010	Valproic acid	It was confirmed that the incidence of congenital malformations in infants correlates positively with VPA concentrations in maternal serum. Therefore, all efforts should be undertaken to reduce fetal exposure to this drug. One option could be a reduction of the placental drug transfer.

Conceptually, the studies noted above would appear to be directed at investigating the role of the placenta in one of the fundamental principles of teratology: the ability of a teratogen to reach the developing embryo (Wilson 1959). For the purpose of this review, it is useful to contextualize that principle with another: that susceptibility to teratogenesis varies with the developmental stage at which exposure to an adverse influence occurs. Since James G. Wilson first published his six principles of teratology in 1959, our understanding of the critical periods of teratogenic sensitivity has been refined by experimental teratology studies conducted using animal models in which the timing of pregnancy and exposure can be readily isolated.

The placenta serves several functions that influence embryonic and fetal development. This review focuses specifically on the role of the placenta in teratogenesis, the process by which malformations result from embryonic exposure to teratogens via maternal circulation, primarily by examining the temporal relationship of teratogen susceptibility and establishment of the hemochorial placenta.

## 2 Critical Periods of Sensitivity to Teratogen‐Induced Structural Malformations

**FIGURE 1:**
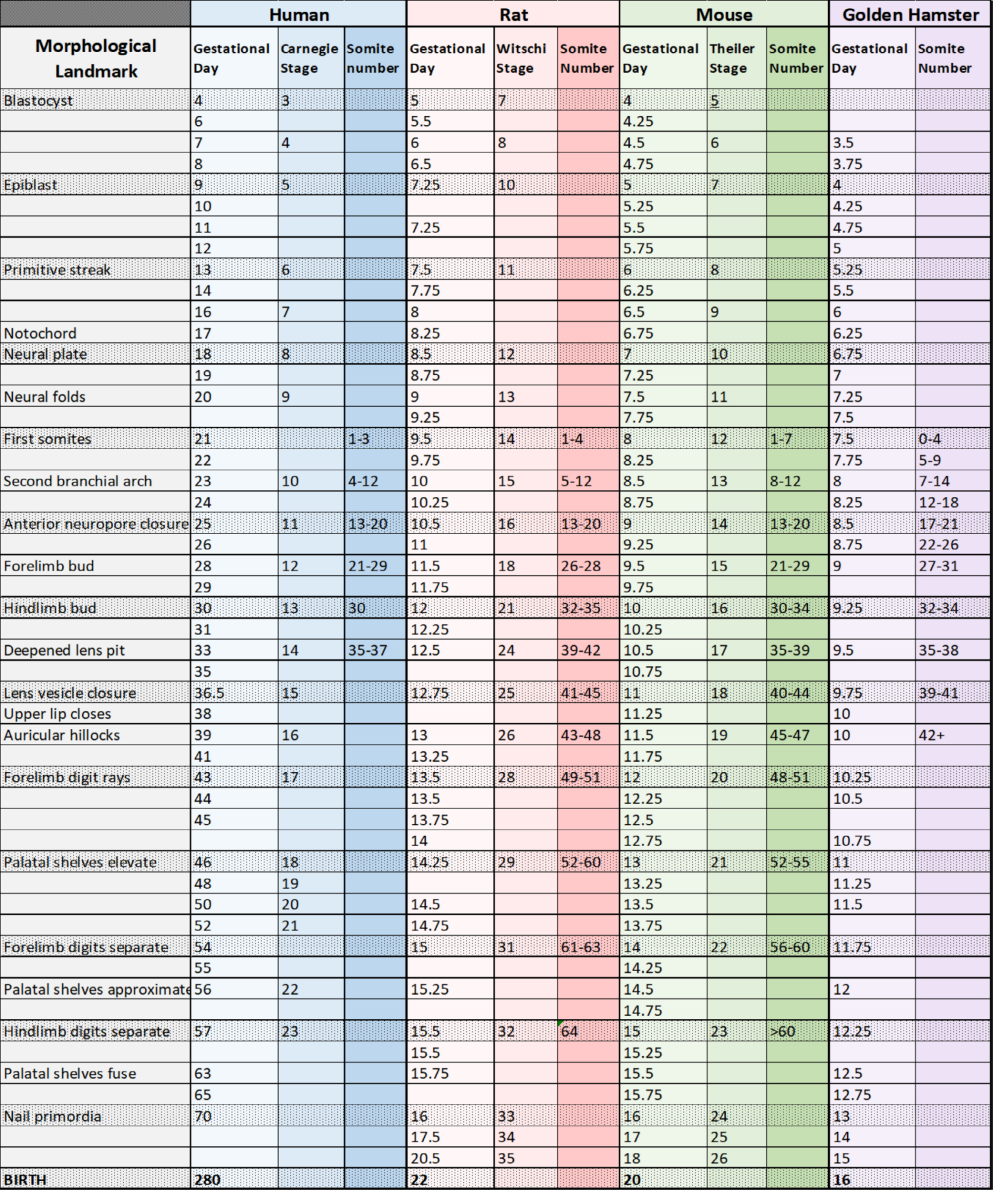
Multi‐species comparative developmental timeline. Developmental timing for human, rat, mouse, and golden hamster aligned by somite number and commonly documented developmental landmarks. Species‐specific gestational days, somite number ranges, and developmental landmarks are also aligned with commonly used schemas for describing human (Carnegie), rat (Witschi), and mouse (Theiler) developmental stages.

To evaluate the relationship between placental maturation and teratogenic susceptibility, we first define the developmental windows of teratogen‐induced malformations. While the molecular, cellular, and morphological mechanisms of embryogenesis are highly conserved among mammals, overall gestational length varies considerably across species. To relate animal model experimental paradigms and findings to each other and to human developmental timing, we developed a multi‐species comparative developmental timeline (Figure 1). We focused on mammalian model studies because of their prevalence and relevance to placental biology. Developmental staging information for human, rat, and mouse was primarily extracted from textbooks (Witschi 1957; Theiler 1989) and reputable online atlases (Hill 2025), while staging information for the golden hamster was extracted from primary research papers (Hoffman et al. 1968; Shenefelt 1972). Alignment of gestational days (GDs) across species was primarily based upon available documentation of somite number, which is widely considered the most precise indicator of developmental stage, and commonly documented developmental landmarks. For the purpose of this review and for general use as a reference, species‐specific gestational days, somite number ranges, and developmental landmarks are listed alongside commonly used schemas for describing human (Carnegie), rat (Witschi), and mouse (Theiler) developmental stages.

We next identified published studies from which teratogen‐induced malformation data could be extrapolated and plotted on the multi‐species developmental timeline. For this purpose, studies in which a single, defined teratogen was administered acutely at a specified time point and external malformations were observed and recorded were prioritized. Focus was placed on overt external malformations, as these would presumably be more likely to be evaluated and recorded than subtle dysmorphic phenotypes or those that require extensive dissection to evaluate. Most suitable studies were conducted in animal models, which allow for precise control of the timing of pregnancy and teratogen administration. While determining timing of exposure is more challenging in humans, the findings of one meta‐analysis that established windows of sensitivity to thalidomide‐induced malformations in humans were also incorporated (Brent and Holmes 1988). The species and teratogen examined in each of the individual studies selected for this analysis are listed in Table 2. Included animal studies follow two general approaches: teratogen administration targeting one or two pre‐determined developmental timepoints (Sulik et al. 1981, 1995; Lipinski et al. 2012; Sun et al. 2021) and those in which a teratogen was administered at regular intervals to different dams across a broader developmental window (Shenefelt 1972; Omnell et al. 1990; Heyne et al. 2015). The latter approach has the advantage of demarcating windows of sensitivity from insensitivity to a given teratogen for a specific malformation outcome.

**TABLE 2:** Selected teratogenicity studies.

Publication	Species	Teratogen	Mechanism
Brent and Holmes (1988)	Human	Thalidomide	Multiple mechanisms proposed
Sulik et al. (1981)	Mouse	Ethanol	Multiple mechanisms proposed
Lipinski et al. (2012)	Mouse	Ethanol	Multiple mechanisms proposed
Omnell et al. (1990)	Mouse	Jervine	Sonic Hedgehog pathway antagonist
Heyne et al. (2015)	Mouse	Vismodegib	Sonic Hedgehog pathway antagonist
Sun et al. (2021)	Mouse	Tamoxifen	Teratogenic mechanism unknown
Sulik et al. (1995)	Mouse	Retinoic acid	Retinoic acid receptor agonist
Shenefelt (1972)	Golden Hamster	Retinoic acid	Retinoic acid receptor agonist

**FIGURE 2:**
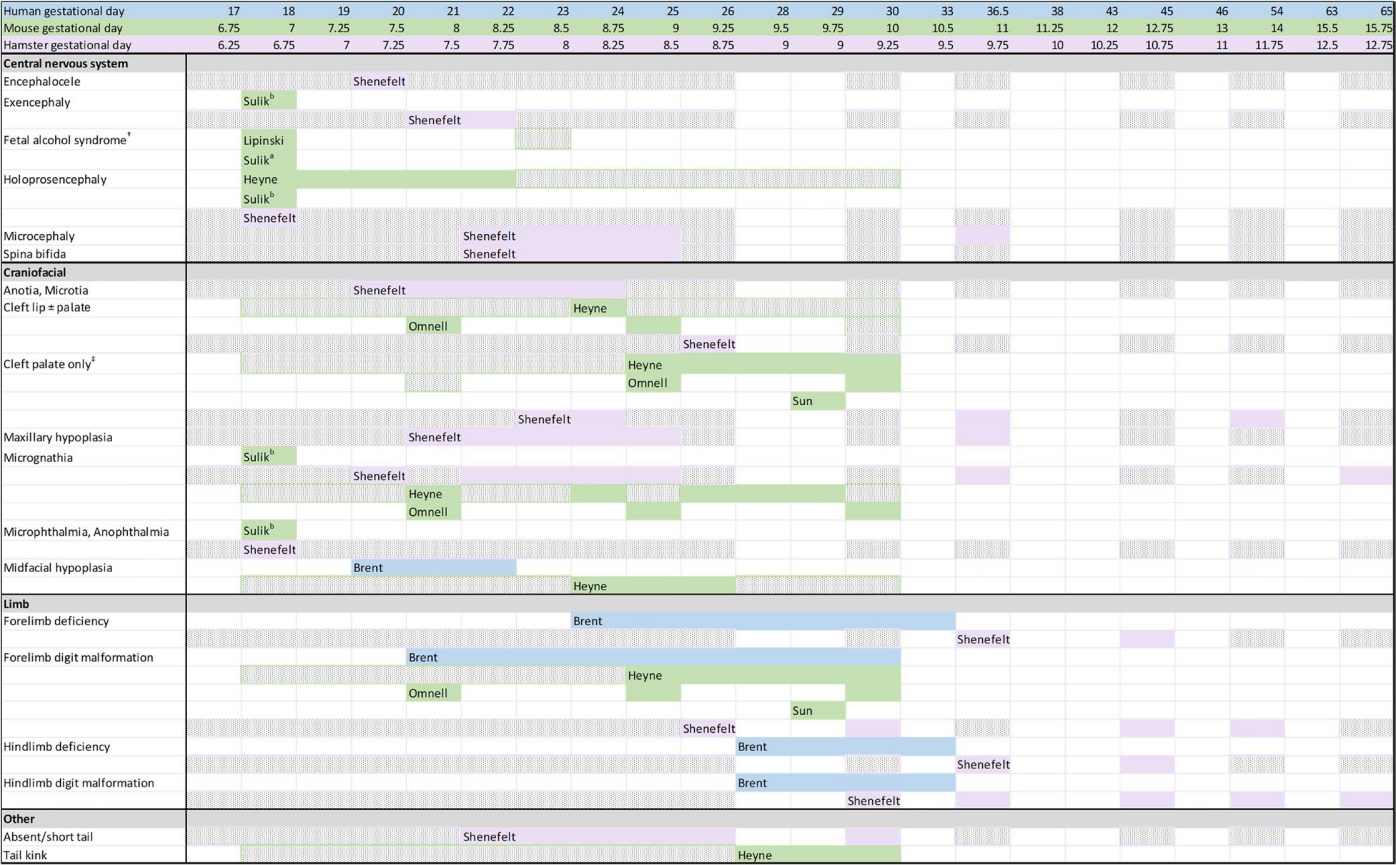
Timeline of critical periods for induction of structural birth defects. Windows of sensitivity versus insensitivity are indicated in this figure by filled versus hatched cells, respectively. ^†^Presence of cardinal craniofacial features of severe fetal alcohol syndrome, including microcephaly, shortened palpebral fissures, and a long, smooth philtrum. ^‡^Cleft palate only versus cleft lip and palate phenotypes could not be confidently delineated from the published material for some studies.

Using mouse developmental timing as a reference for major trends, Figure 2 illustrates that central nervous system malformations have the earliest window of sensitivity, arising primarily from teratogenic exposure between GD7 and GD9. Reflecting both the sequencing and interrelationship of development of the brain and face, the critical periods for craniofacial malformations overlap with, and extend beyond, those for the central nervous system. The critical periods for induction of limb malformations cluster later in development, yet nearly all have critical periods of sensitivity at or before GD10. The window of sensitivity for forelimb malformations preceding that for hindlimbs is consistent with the progressive sequence of fore‐ and hindlimb bud emergence. Accordance of critical windows between animal models and humans is supported by the overlap of malformations ascribed to thalidomide exposure in humans and those induced by other teratogens in animal models, as well as studies placing the general window of sensitivity for alcohol and retinoic acid teratogenesis in the first trimester in humans (Ernhart et al. 1987; Day et al. 1989; You et al. 2024). GD7–11 in the mouse corresponds to human GD18–38, such that the critical window for most teratogen‐induced malformations translates to approximately the 3rd to the 6th week of human development. This interval is consistent with previous estimations that teratogens have their major impact upon the structural form of a developing embryo during the first 8 weeks of development (Finnell et al. 2002).

## 3 Placental Development

To evaluate the placenta's role as a barrier during critical periods of teratogen sensitivity, we next outline the timing of placental maturation.

## 4 Mouse Placental Development

We initially focus here on mouse, both because the above‐described teratogenicity data were predominantly generated using this species and because our understanding of the timing of placental development in mice is more precise than that in humans. Murine placental development has been described elsewhere, including one recent and particularly informative review (Panja and Paria 2021). Development of the placenta in mice is a highly orchestrated process that establishes a sophisticated biological interface for regulated exchange between the mother and the fetus by gestational day 10 (GD10).

Before GD10, the embryo is not yet supported by a fully functional placenta and relies on a combination of physical barriers and nutritional mechanisms that are less well‐defined in terms of their effectiveness in protecting against maternal toxins and toxicants. During the first 3–4 days of development, the preimplantation embryo is surrounded by the zona pellucida (ZP), a glycoprotein envelope that physically separates the embryo from maternal tissues. The ZP acts as a physical barrier, preventing direct contact between the embryo and maternal tissues or fluids. The embryo remains suspended within the lumen of the oviduct and subsequently the uterus. After the blastocyst hatches from the ZP and implants into the uterine wall (around GD4.5), a series of new structures develop. The outer layer of the blastocyst, the trophoblast, forms the primary barrier between the embryo and maternal tissues. These cells play a critical role in initiating implantation and consequent placental development and regulating the exchange of substances. The uterine stromal cells transform into decidual cells, forming two distinct zones, the Primary Decidual Zone (PDZ), which is established by GD6 and acts as the first protective scaffold for the implanted embryo, and the Secondary Decidual Zone (SDZ), which surrounds the PDZ and provides additional protection and support. In mice, the yolk sac envelops the embryo and serves as an important barrier and nutritional interface. The yolk sac's extraembryonic endoderm absorbs nutrients through active pinocytosis and transfers them to the embryo. The yolk sac also acts as a barrier, but its ability to protect against environmental factors in the maternal circulation is unclear.

During organogenesis, the developing embryo is supported not only by emerging maternal blood flow through placental villi, but also by two parallel extra‐embryonic transport systems that are active long before the hemochorial placenta is established. First, the vitelline circulation connects the embryo's primitive dorsal aorta to the visceral yolk sac (VYS), where a rich capillary plexus absorbs nutrients, gases, and macromolecules—including environmental toxins—and returns them via vitelline veins to the embryo. In rodents, this circulation is fully functional by GD8.5 and remains the primary route of embryonic nutrition until mid‐gestation, overlapping precisely with critical windows for teratogen‐induced structural malformations (GD7–GD10). Second, the umbilical circulation begins developing concomitantly, establishing early exchange between the embryo and allantoic mesoderm before definitive placental labyrinth formation. Because both circulatory routes are capable of delivering xenobiotics, teratogens can access the embryo via yolk sac pinocytosis or early trophoblast transport well before maternal blood perfuses the mature placenta.

The VYS serves as an initial functional placenta mediating early embryonic vulnerability. In rodents, the VYS persists throughout gestation as an inverted, highly pinocytic epithelium that absorbs teratogens through receptor‐mediated endocytosis, degrades them within lysosomes, and transfers products to the embryo. Classic studies demonstrate that antibody‐mediated inhibition of yolk sac pinocytosis induces malformations without direct embryonic exposure, confirming yolk sac dysfunction as intrinsically teratogenic (Brent 1966; Freeman et al. 1982). Finally, histotrophic nutrition via uterine glands persists until week 8 in humans, and first‐trimester placental explants (6–14 weeks) exhibit active small‐molecule accumulation, metabolic processing (e.g., hCG synthesis), and bioactivation of toxins (Genbacev et al. 1992; Miller et al. 2005). Collectively, pre‐placental structures provide both protective and permissive pathways for teratogens during organogenesis.

By GD10, the mouse placenta has developed into a complex, multi‐layered structure that effectively juxtaposes the maternal and fetal circulations without mixing. The placenta becomes structurally mature by GD15 and achieves maximum volumetric growth by GD17. The three main layers of the placenta—the labyrinth zone, junctional zone, and maternal decidua—work in concert to create a protective yet permeable interface. The labyrinth zone is the primary site of maternal‐fetal exchange and consists of four distinct cell layers: sinusoidal trophoblast giant cells (S‐TGCs) that face maternal blood sinusoids and act as the first line of contact with maternal blood, syncytiotrophoblast (SynT‐I and SynT‐II) layers that form the middle barrier and are critical for regulating the passage of substances between maternal and fetal circulations, and fetal endothelial cells that line fetal blood vessels and complete the barrier, ensuring that only select substances can pass through. The intermediate junctional zone layer (p‐TGC) regulates the entry of maternal substances into the fetal portion of the placenta. The p‐TGC layer borders the decidua and plays a key role in controlling what passes through to the labyrinth zone. The maternal decidua is the outermost layer of the placenta and provides the first line of defense by regulating the initial entry of substances from maternal circulation into the placenta.

## 5 Human vs. Mouse Placental Development

The developmental timeline and structural makeup of the mouse placenta provide a valuable framework for understanding human placental development, though key differences in timing and anatomy must be emphasized. Efforts to elucidate the details of human placental development have been benefited by new in vitro and in vivo studies, the results of which have been described in a recent review paper (Hemberger et al. 2020). In humans, the ZP persists until implantation at day 6–7 post‐fertilization (equivalent to mouse GD4.5). The blastocyst implants earlier relative to gestational age, with trophoblast differentiation initiating shortly after. Chorionic villi begin forming by week 3, and the hemochorial placenta—where maternal blood directly bathes trophoblasts—is established by week 8–10. The yolk sac regresses earlier in humans (by ~12 weeks), but remains critically active during weeks 4–6—the peak window of structural teratogen susceptibility; impaired morphology correlates with adverse outcomes in high‐risk pregnancies Ornoy and Miller 2023).

The mouse labyrinth zone (GD10 onward) mirrors the human villous tree, which develops from week 8. Both structures mediate maternal‐fetal exchange through syncytiotrophoblasts and fetal endothelial cells. In humans, the villous barrier is trilaminar (syncytiotrophoblast, cytotrophoblast, fetal endothelium), whereas mice have a tetralaminar labyrinth. Mouse decidual zones (PDZ/SDZ) and junctional zone (p‐TGCs) have parallels in the human decidua basalis and extravillous trophoblasts. These layers regulate immune tolerance and limit invasive trophoblast migration in humans.

Both species achieve functional maternal‐fetal juxtaposition by mid‐gestation (~GD10 in mice, ~week 10 in humans). However, human placental maturation continues until term, while the mouse placenta peaks in volume by GD17 (equivalent to human week 38). The critical period of placental vulnerability in mice (pre‐GD10) aligns with human weeks 2–8, when organogenesis occurs and placental barriers are immature. Post‐GD10 in mice (~human week 8 onward), the placenta assumes a more protective role, though some toxins and toxicants still cross the labyrinth/villous barrier and impact fetal development.

## 6 Discussion

In this review, we demonstrate that the peak period of teratogen sensitivity precedes the functional maturation of the placenta, challenging the assumption that placental transfer is a primary determinant of teratogenic risk during organogenesis. In the mouse, each of the malformations assessed could be induced by teratogen exposure between GD7 and GD10, while the mouse hemochorial placenta is established at GD10 and becomes fully functional at GD15. The critical periods for most teratogen‐induced structural birth defects, as defined by studies in mouse and golden hamster, translated to the 3rd to 6th week of human embryonic development, consistent with available human teratogenicity evidence. The human hemochorial placenta develops between the 8th and 12th week of development. This temporal discordance suggests that structural malformations arise during a developmental window when embryonic susceptibility is high and placental protection from toxin/toxicant transfer to the embryo is minimal.

The focus of this review is on structural malformations that result from disruption during the organogenesis phase of embryonic development. However, it is important to recognize that functional anomalies like neurodevelopmental and metabolic disorders have long been recognized to have broader windows of susceptibility that extend beyond organogenesis and into fetal development. One relevant example is the comparison of fetal alcohol syndrome with the broader constellation of fetal alcohol spectrum disorder. While the salient features of full‐blown fetal alcohol syndrome, including microcephaly and facial dysmorphology, are well recognized to result from ethanol exposure early in embryogenesis (i.e., GD7.0 in mice), ethanol exposure later in development can result in neurodevelopmental deficits in the absence of overt dysmorphology (Lipinski et al. 2012; Almeida et al. 2020). Even some structural malformations like hypospadias have critical periods later in embryonic development when the hemochorial placenta is established (Clark et al. 1993). It is also important to recognize that certain congenital conditions like those associated with intrauterine growth restriction are thought to arise from placental dysfunction.

While this analysis focused explicitly on the timing and capacity of placental transfer, placental biology encompasses multiple additional mechanisms that can also modulate teratogenic and/or birth defect risk. Teratogens may disrupt placental vascularization, altering nutrient and oxygen delivery; interfere with the syncytiotrophoblast's endocrine functions—such as hCG and steroid hormone synthesis—that regulate maternal–fetal homeostasis; or perturb placental metabolic enzymes responsible for xenobiotic biotransformation. Each of these processes can indirectly impact the embryo even when direct transfer is minimal or delayed. However, the critical window for structural malformations coincides with a period of minimal maternal blood perfusion of chorionic villi, underscoring that transfer‐dependent exposure is unlikely to be the dominant driver of most birth defects during early organogenesis.

Accordingly, we propose that future investigations should decouple assessments of placental transfer kinetics from studies of placental dysfunction. Quantitative analyses of small‐molecule permeability should be complemented by mechanistic studies of trophoblast and vascular integrity, endocrine signaling, and metabolic capacity. Such a multifaceted approach will resolve how each placental function contributes to embryonic vulnerability and will clarify whether alterations in placental physiology—rather than transfer per se—indirectly mediate teratogenic effects at later stages of gestation.

Prior to maturation of the true placenta, the embryo is surrounded by a combination of structures, including the zona pellucida, trophoblast, and yolk sac (collectively termed the “gestational sac”). However, the effectiveness of these barriers to prevent environmental factors in maternal circulation from reaching the developing embryo is poorly understood (Koren and Ornoy 2018). Several scenarios have been proposed for passive and/or active transport of substances into and out of the gestational sac (Adibi et al. 2021), but there is minimal primary evidence from which to directly evaluate these proposed mechanisms. We can, however, make key inferences from teratology studies and complementary investigations of teratogenic mechanisms. For instance, that the substances described above (ethanol, retinoic acid, valproic acid, Sonic hedgehog signaling antagonists) are teratogenic when administered early in embryogenesis suggests that they readily reach the embryo. Whilean impact on the gestational sac itself has been proposed as a possible mode of teratogenicity, most teratogens have been found to directly target embryonic molecular mechanisms that regulate morphogenesis, suggesting direct teratogenic action on the embryo itself.

## 7 Implications for Teratology Research

Whether the gestational sac effectively protects against other would‐be teratogens is poorly understood, particularly in human development. As cell‐based and in silico high‐throughput approaches are increasingly used to identify potential environmental birth defect risk factors, a better understanding of whether and how pre‐placental structures act as a barrier for certain substances is increasingly important. As recognized by James G. Wilson, teratogenicity is dependent upon the ability of the substance to reach the embryo. Future efforts to understand how known teratogens act, as well as those to predict and assess additional environmental birth defect risk factors, should be informed by an understanding of the structures that surround and protect the embryo during the critical period of teratogenic sensitivity. For example, investigations of teratogen‐induced malformations should prioritize the embryonic microenvironment and gestational sac dynamics during early organogenesis.

## Conflicts of Interest

The authors declare no conflicts of interest.

## Data Availability

The data that support the findings of this study are available from the corresponding author upon reasonable request.
